# Restoration of the anterior compartment after robotic total knee arthroplasty significantly improves functional outcome and range of motion at 1 year

**DOI:** 10.1002/ksa.12337

**Published:** 2024-06-27

**Authors:** Moussa Kafelov, Cécile Batailler, Elvire Servien, Sébastien Lustig

**Affiliations:** ^1^ Orthopaedics surgery and Sports Medicine Department FIFA Medical Center of Excellence, Croix‐Rousse Hospital, Lyon University Hospital Lyon France; ^2^ IFSTTAR, LBMC UMR_T9406 Univ Lyon, Université Claude Bernard Lyon 1 Villeurbanne France; ^3^ University Multiprofile Hospital for Active Treatment and Emergency Medicine ‘N. I. Pirogov’ Bulgaria; ^4^ Interuniversity Laboratory of Biology of Mobility, LIBM—EA 7424 Claude Bernard Lyon 1 University Lyon France

**Keywords:** anterior compartment restoration, functional outcomes, functional positioning principles, image‐based robotic‐assisted system, total knee arthroplasty

## Abstract

**Purpose:**

This study aims to assess the functional outcomes based on restoring the anterior compartment after total knee arthroplasty (TKA).

**Methods:**

This retrospective study included 96 primary TKAs performed between 2021 and 2022. Functional positioning principles were applied using an image‐based robotic‐assisted system. The mean age was 69.2 ± 7.9 years. Knee Society Score (KSS), Kujala score, Forgotten Joint Score (FJS) and knee flexion were collected preoperatively and at 1 year. The depth difference between native and prosthetic trochlea was measured to assess anterior compartment restoration at full extension, 30°, 70° and 90° flexion. The global anterior compartment restoration combined the anterior compartment restoration and the patellar thickness restoration.

**Results:**

The trochlear offset was mostly understuffed after TKA compared to the native anatomy, mainly for medial and lateral condyles at 30° and 70° of flexion. The global anterior compartment restoration was understuffed in full extension (−0.7 mm ± 2), at 30° (−4.4 mm ± 2) and 70° of flexion (−3.6 mm ± 2.5). At 90°, the global anterior compartment restoration was overstuffed (2.2 mm ± 1.8). Functional scores were not significantly influenced by the anterior compartment stuffing at 0° and 30° (n.s.). The anterior compartment overstuffing at 70° and 90° was associated with decreased KSS function score (*p* = 0.009) and flexion (*p* = 0.04).

**Conclusion:**

Moderate anterior understuffing was frequently observed after TKA performed with functional positioning and an image‐based robotic‐assisted system. This understuffing did not influence the functional outcomes. The overstuffing of the anterior compartment led to a reduction in KSS function score and flexion measurements at 1 year.

**Level of Evidence:**

Level III, retrospective cohort study.

**Abbreviations**
BMI: body mass index
FKJS: Forgotten Joint Score
HKA: hip–knee–ankle angle
JLCA: joint line congruency angle
JLO: joint line obliquity
KSS: Knee Society Score
LDFA: lateral distal femur angle
MPTA: medial proximal tibial angle
PCA: posterior condylar axis
PROMs: patient‐reported outcome measures
PS: posterior stabilised
RAS‐FA: robotic‐assisted functional alignment
RA‐TKA: robotic‐assisted total knee arthroplasty
TEA: transepicondylar axis
TKA: total knee arthroplasty

## INTRODUCTION

Restoration of the anterior knee compartment during total knee arthroplasty (TKA) is being studied increasingly [3, 10] with the development of personalised surgery. Even little imperfections of the anterior compartment restoration can lead to patellar maltracking, severe pain and poor functional outcomes [17]. The patellar tracking is dictated by surgical techniques (notably, implant positioning) and implant characteristics (trochlear depth and shape, sagittal curvature and patellar component design) [5, 34]. The design of the prosthetic trochlea differs from that of the native one [28]. The perfect restoration of the anterior compartment is thus compromised, even with personalised surgical technique [27].

Conventional jig instrumentation is limited for the restoration of the anterior compartment. The posterior reference is accurate for resectioning posterior condyles but less for the anterior compartment [6, 8]. The femoral rotation impacts the trochlear restoration and can be determined using several techniques. However, their accuracy is variable [14, 19]. New robotic‐assisted technologies allow surgeons to unpair patellofemoral and tibiofemoral compartments and reconsider thinking about the anterior knee compartment. Indeed, thanks to the preoperative computed tomography (CT) scan, the shape of the trochlea is materialised on the screen during the planning. The femoral implant can be superimposed and positioned precisely in the same position as the native trochlea. Consequently, recreating the physiologic position and depth of the trochlea groove appears easier with this robotic assistance. It could significantly impact the patellar tracking and the extensor mechanism function after TKA [11, 32]. Few studies have correlated the restoration of the anterior compartment, particularly, its stuffing, and the functional outcomes after TKA [13].

This study aimed to evaluate (1) the restoration of the trochlea offset after primary TKA, performed with an image‐based robotic‐assisted system, (2) the restoration of the anterior compartment, including the patella thickness, after TKA and (3) the functional outcomes based on the restoration of the anterior compartment at 12 months after TKA. The hypothesis was that restoring the anterior compartment impacts the functional outcomes after TKA.

## MATERIALS AND METHODS

**Figure 1:**
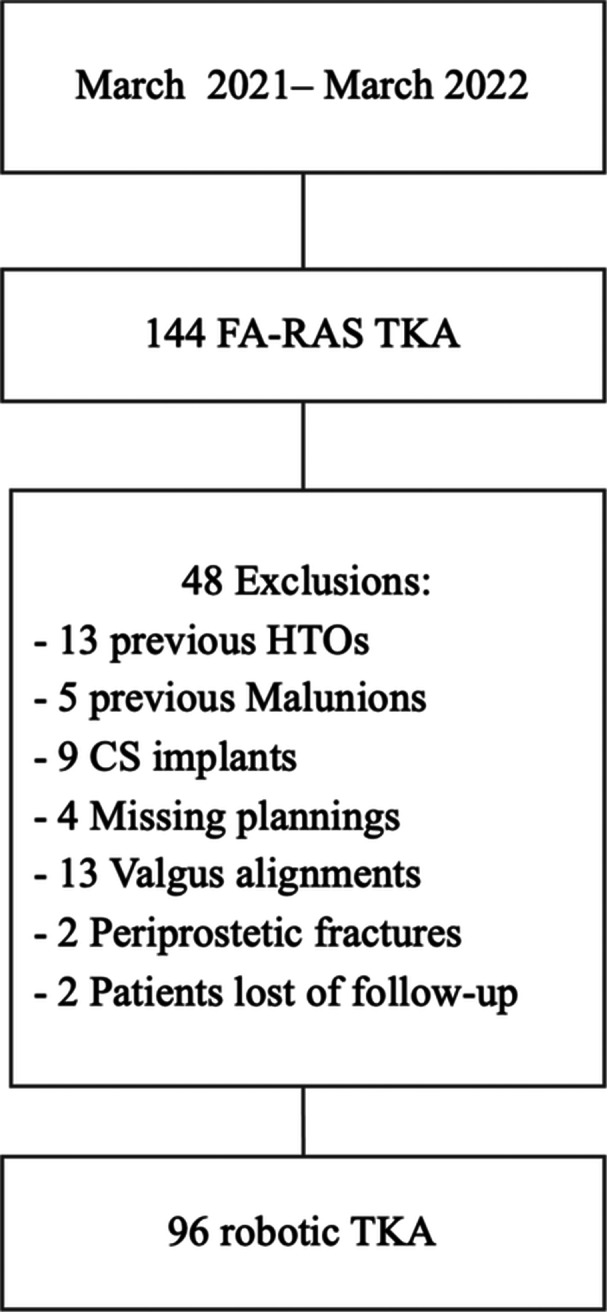
Flowchart. AP, antero‐posterior; CS, cruciate substituting; FA‐RAS, robotic‐assisted functional alignment; HTO, high tibial osteotomy; TKA, total knee arthroplasty.

This retrospective single‐centre study included 96 consecutive patients who underwent primary image‐based robotic‐assisted TKA (MAKO, Stryker Corp.) for end‐stage varus osteoarthritis between March 2021 and 2022 with a 1‐year minimum follow‐up. The exclusion criteria were revision cases, previous femoral osteotomy, posttraumatic femoral osteoarthritis and preoperative valgus alignment on weight‐bearing X‐rays (hip–knee–ankle angle [HKA] > 183°). One hundred forty‐four primary TKAs were performed by an image‐based robotic‐assisted system applying the functional alignment principles [31] during this period. After using exclusion criteria, 96 TKA were evaluated for final analysis (Figure 1).

Two high‐volume arthroplasty surgeons performed all surgeries. Standardised functional positioning principles [31] and the medial subvastus approach were used in all cases. A tourniquet was not applied. The implant was the same for all the patients: posterior‐stabilised TKA (Triathlon PS, Stryker Corp.).

MAKO Total Knee planning software (Stryker) built personalised three‐dimensional knee models based on preoperative supine CT scans. The Triathlon PS implant was positioned on this model based on the functional positioning principles [31]. Accuracy within 1 mm between the implant planning and final position is already proven [33]. The patella was resurfaced in cases of anterior knee pain, patellofemoral osteoarthritis (Iwano ≥ 2), inflammatory arthritis and crystalline arthropathy.

CT scan and X‐ray series, including anteroposterior, lateral in 30° flexion, weight‐bearing long leg film and skyline view, were done preoperatively for every patient. All radiographic measurements were made using PACS software (Centricity™, GE Healthcare). The HKA [24], the patellar height with the Blackburne index [2] and the patellar tilt were measured using standardised techniques. Knee Society Score (KSS) (knee and function scores) [7], Kujala score, Forgotten Joint Score (FJS), anterior knee pain and knee flexion were collected preoperatively and at 1 year of follow‐up.

The restoration of the trochlear offset and the patellar thickness have been calculated to assess the restoration of the anterior compartment.

**Figure 2:**
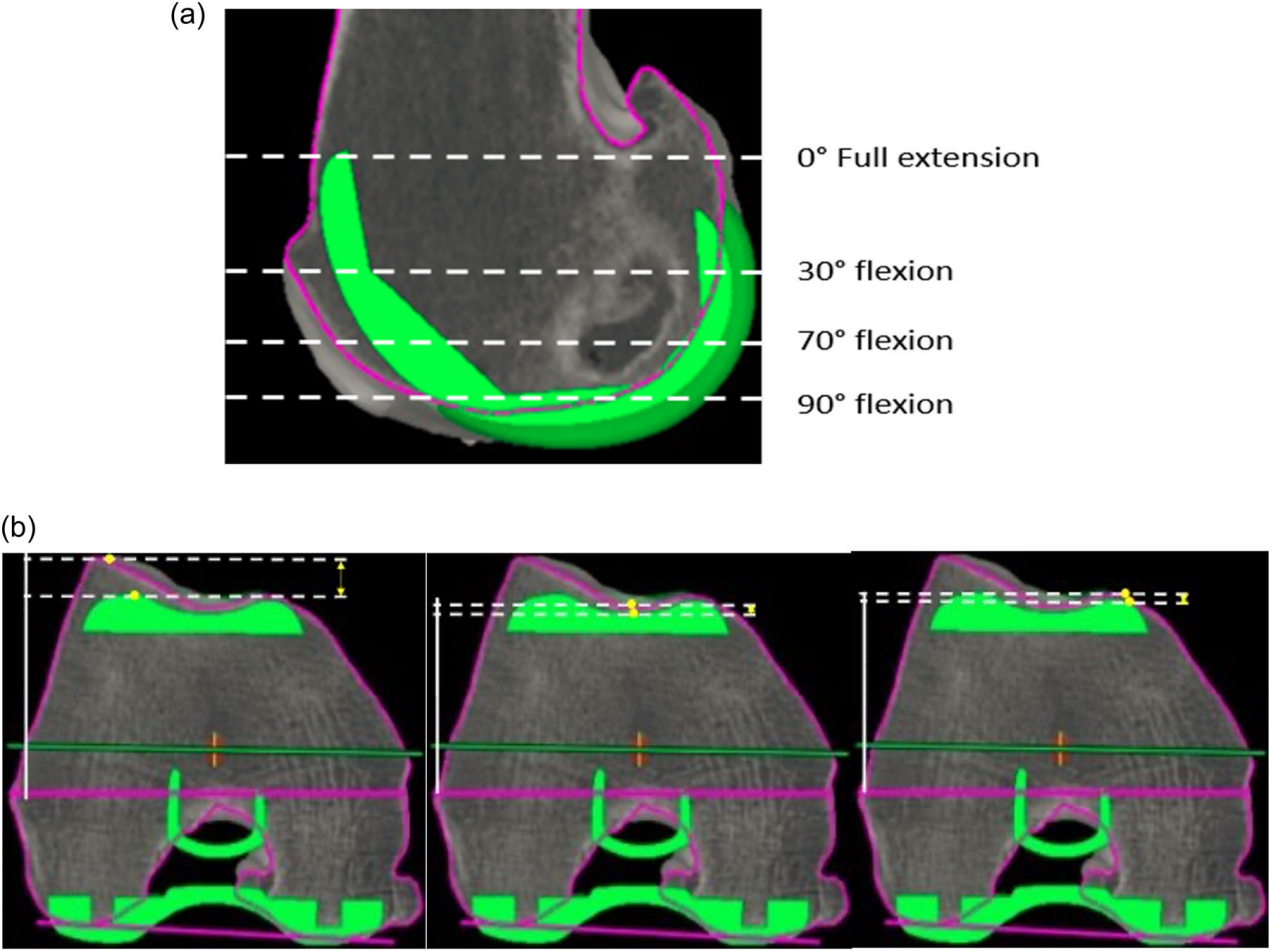
(a) Measurement method—the difference between the native bone and the prosthesis was measured in four positions: full extension (0°), at 30° flexion, at 70° flexion and in full flexion (90°). For each of these positions, we choose an axial slice with the following markings: the first axial slice with a clearly defined prosthetic trochlear groove (‘full extension’), the second was the merging point of the anterior chamfer and anterior flange on the sagittal view (‘at 30° flexion’), the third one is the last slide on which both the prosthetic groove and femoral groove were visible (‘at 70° flexion’), the fourth was the position on which the anterior flange and distal femoral part met (‘at 90° flexion’). (b) Measurement of the distance between the highest point on the prosthesis and the highest point on the patient's native bone at 30° of flexion (lateral condyle, trochlea groove, then medial condyle).

An already described and validated measurement technique was used for the trochlear offset [32]. All measurements were performed using MAKO robotic platform planning software (Mako, Stryker Corp.). A calibrated scale in millimetres allowed accurate and reliable measurements, with an accuracy of 1 mm. The measurements of the restoration of the anterior compartment after TKA were performed by one independent orthopaedic surgeon (M. K.). We measured the restoration of the trochlear offset in several positions in the medial, lateral and central parts of the trochlea. As described previously, four sequential positions were assessed where the patella is engaged in the femoral groove with knee flexion (Figure 2) [12, 32]. For each of these positions, we choose an axial slice with the following markings: the first axial slice with clearly defined prosthetic trochlear groove (‘full extension’), the second was the merging point of the anterior chamfer and anterior flange on the sagittal view (‘at 30° flexion’), the third one is the last slide on which both the prosthetic groove and femoral groove were visible (‘at 70° flexion’), the fourth was the position on which the anterior flange and distal femoral part met (‘at 90° flexion’). For each of these positions, we compared the highest point of the lateral native condyle and the lateral prosthetic condyle, the highest point of the medial native condyle and the medial prosthetic condyle, the deepest end of the native trochlear groove and the prosthetic trochlea (Figure 2). For these measurements, the reference line used was the transepicondylar axis.

For the patellar thickness, we subtract the original patellar thickness (measured intraoperatively with a caliper with an accuracy of 1 mm) from the combined thickness of the bone after resection and the thickness of the patellar button. The value was positive if the combined thickness was superior to the original and negative if the combined thickness was inferior to the original. When the patella was not resurfaced, the value was 0 (no change of initial thickness). The patellar button was symmetrical.

Patellar thickness difference=Native patellar thickness−(Polyimplant+Thickness after resection).

To determine the global anterior compartment restoration at each degree of flexion, we used the following formula based on the difference between native bone and femoral implant of the lateral condyle (bone lateral: bL; implant lateral: iL), the medial condyle (bone medial: bM; implant medial: iM) and the trochlear groove (bone trochlea: bT; implant trochlea: iT).

$$\mathrm{Global}\;\mathrm{anterior}\;\mathrm{compartment}\;\mathrm{restoration}=\frac{\left(\mathrm{bL}-\mathrm{iL}\right)+\left(\mathrm{bM}-\mathrm{iM}\right)+\left(\mathrm{bT}-\mathrm{iT}\right)}{3}+\mathrm{Patellar}\;\mathrm{thickness}\;\mathrm{difference}.$$

All statistical analysis was performed using XLSTAT (2021, Addinsoft). Continuous variables were described using means, standard deviation and ranges. Categorical variables were described using counts (percent). An univariate analysis and then the multinomial logistic regression model were used to evaluate the correlations between functional outcomes and the restoration of the anterior compartment. The correlations between functional outcomes and the restoration of the anterior compartment were also assessed by the Kruskal–Wallis test, with categories of under and overstuffing (every 5 mm). The significance threshold was set at 5%. A post hoc power analysis was performed on the KSS, flexion and Kujala scores. This post‐hoc power analysis found a power between 83% and 87%.

## RESULTS

The mean age was 69.2 ± 7.9 years [50; 86], and the mean BMI was 29.8 kg/m^2^ ± 4.7 [20.7; 42.1]. Patients were equally distributed by gender (male, *n* = 48 (50%)). The preoperative functional scores are reported in Table 1.

**Table 1:** Clinical and radiological outcomes preoperatively and at 1 year.

	Preoperative, *n* = 96	Postoperative, *n* = 96	Difference post–pre
KSS Knee score (mean ± SD) [min; max]	65.3 ± 13.5 [29; 94]	90.7 ± 11.4 [47; 100]	25.6 ± 17.9 [−33; 65]
KSS Function score (mean ± SD) [min; max]	64.2 ± 17.1 [5; 100]	91.1 ± 12.2 [35; 100]	27.3 ± 19 [−30; 95]
FJS score (mean ± SD) [min; max]	NA	74.7 ± 24.7 [18; 100]	NA
Kujala score (mean ± SD) [min; max]	NA	78.1 ± 20 [2; 100]	NA
Flexion (mean ± SD) [min; max]	117.5 ± 12.5 [90; 140]	124.1 ± 8.8 [100; 140]	6.6 ± 14.6 [−30; 50]
Blackburne Index (mean ± SD) [min; max]	0.71 ± 0.19 [0.41; 1.3]	0.69 ± 0.19 [0.20; 1.2]	0.02 ± 0.04 [−0.23; 0.51]
Patellar tilt (mean ± SD) [min; max]	2.6 ± 3.5 [0; 15]	1.9 ± 3.0 [0; 10]	0.51 ± 1.2 [−10; 10]

Abbreviations: FJS, Forgotten Joint Score; KSS, Knee Society Score.

### Trochlear offset restoration

**Figure 3:**
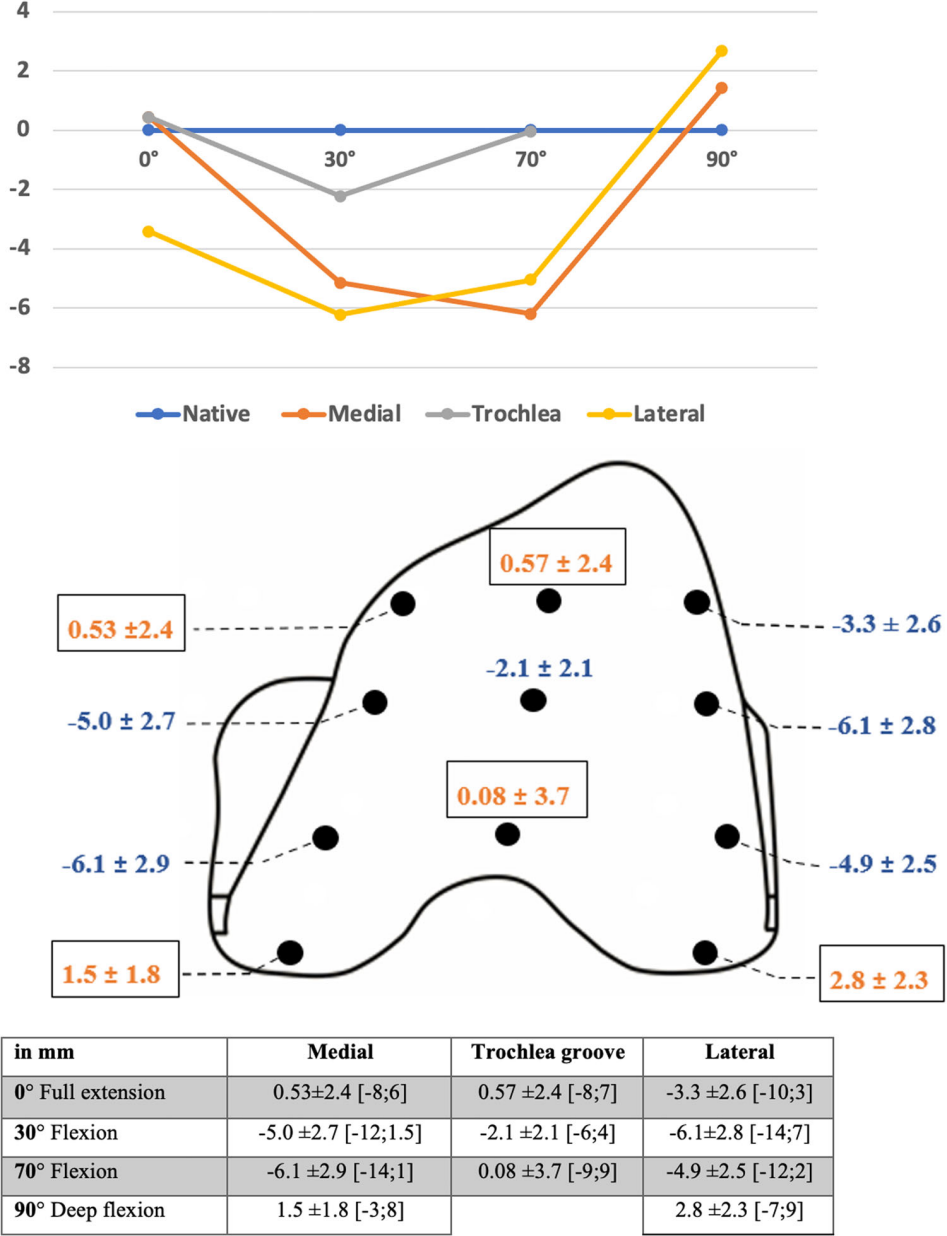
Mean restoration of the trochlear offset (difference between native bone and femoral implant) according to the knee flexion and the localisation in the trochlea (Graphs, localisation on the implant and table).

**Figure 4:**
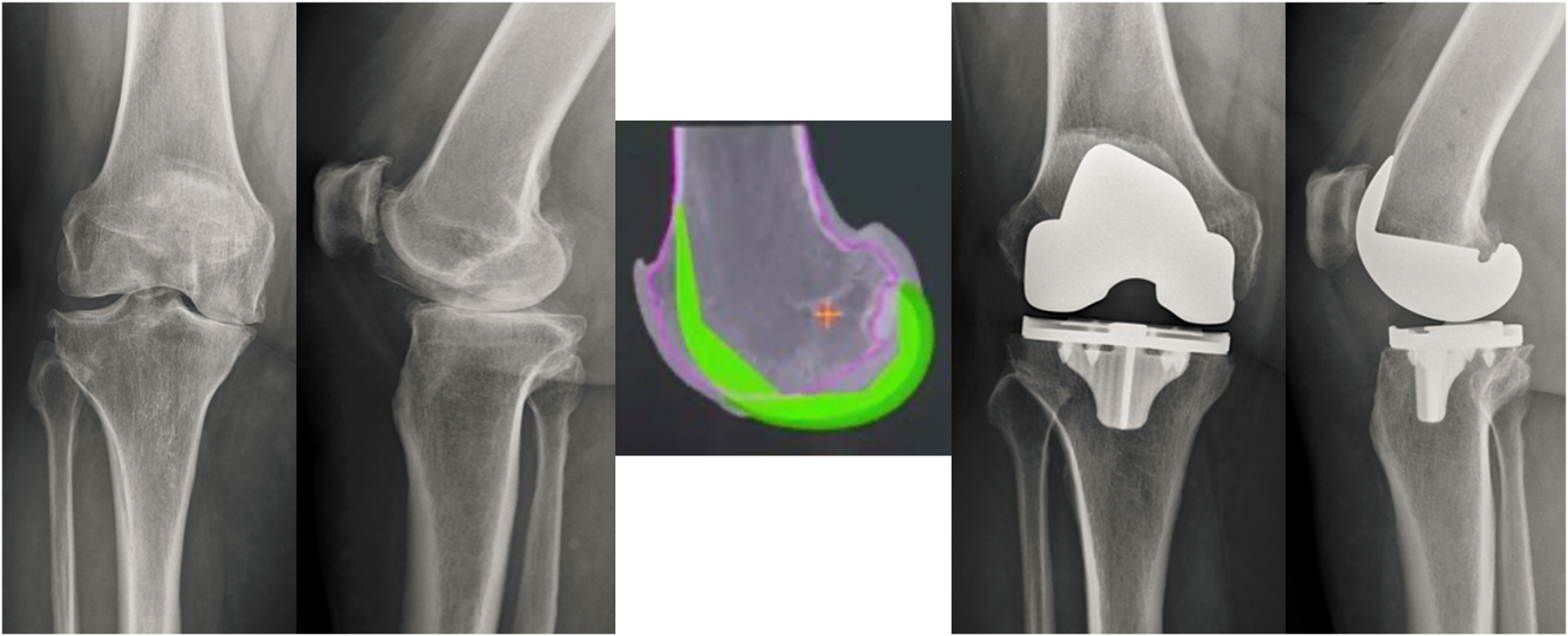
A 70‐year‐old woman with anterior compartment understuffing by trochlear understuffing at 30° and 70° of flexion. Preoperative and postoperative radiographs (AP and lateral views) and robotic planning in the sagittal plane.

The trochlear offset was mostly understuffed after TKA compared to the native anatomy, particularly, at 30° and 70° of flexion (more than 4 mm of understuffing) (Table 2, Figures 3 and 4). The overstuffing was inferior to 2 mm, except for the lateral condyle at 90° of flexion (Table 2).

**Table 2:** Distribution of the trochlear offset restoration according to the localisation on the implant and the knee flexion.

	<−10	−10 to −5	−5 to 0	0–5	>5
0 B/I Med	0	3 (3%)	52 (54%)	40 (41%)	1 (1%)
0 B/I Lat	1 (1%)	26 (27%)	8 (8%)	61 (63%)	0
0 B/I Tr	0	3 (3%)	52 (54%)	37 (38%)	4 (4%)
30 B/I Med	5 (5%)	51 (53%)	38 (40%)	0	2 (2%)
30 B/I Lat	5 (5%)	68 (71%)	1 (1%)	21 (22%)	1 (1%)
30 B/I Tr	0	13 (14%)	11 (11%)	72 (75%)	0
70 B/I Med	12 (13%)	56 (58%)	3 (3%)	25 (26%)	0
70 B/I Lat	4 (4%)	48 (50%)	2 (2%)	42 (44%)	0
70 B/I Tr	0	10 (10%)	29 (30%)	48 (50%)	9 (9%)
90 B/I Med	0	0	66 (69%)	27 (28%)	3 (3%)
90 B/I Lat	0	1 (1%)	74 (77%)	8 (8%)	13 (14%)

Abbreviations: B/I, difference native bone and femoral implant; Lat, lateral condyle; Med, medial condyle; TR, trochlea groove.

### Global anterior compartment restoration

The mean global anterior compartment restoration (including mean trochlear offset restoration in medial, lateral and groove position combined with patellar thickness difference) was understuffed in full extension (−0.7 mm ± 2), at 30° and 70° of flexion (−4.4 mm ± 2 and −3.6 mm ± 2.5, respectively). At 90°, the global anterior compartment restoration was overstuffed (2.2 mm ± 1.8) (Table 3).

**Table 3:** Global anterior compartment restoration according to the degree of knee flexion.

Global anterior compartment restoration (mm)	Mean ± SD [min; max]
In full extension (0°)	−0.7 ± 2 [−6.8; 3.8]
At 30° flexion	−4.4 ± 2 [−8.6; 1.6]
At 70° flexion	−3.6 ± 2.5 [−9.0; 2.7]
At 90° flexion	2.2 ± 1.8 [−5.0; 7.2]

### Correlation between functional outcomes and restoration of anterior compartment

**Figure 5:**
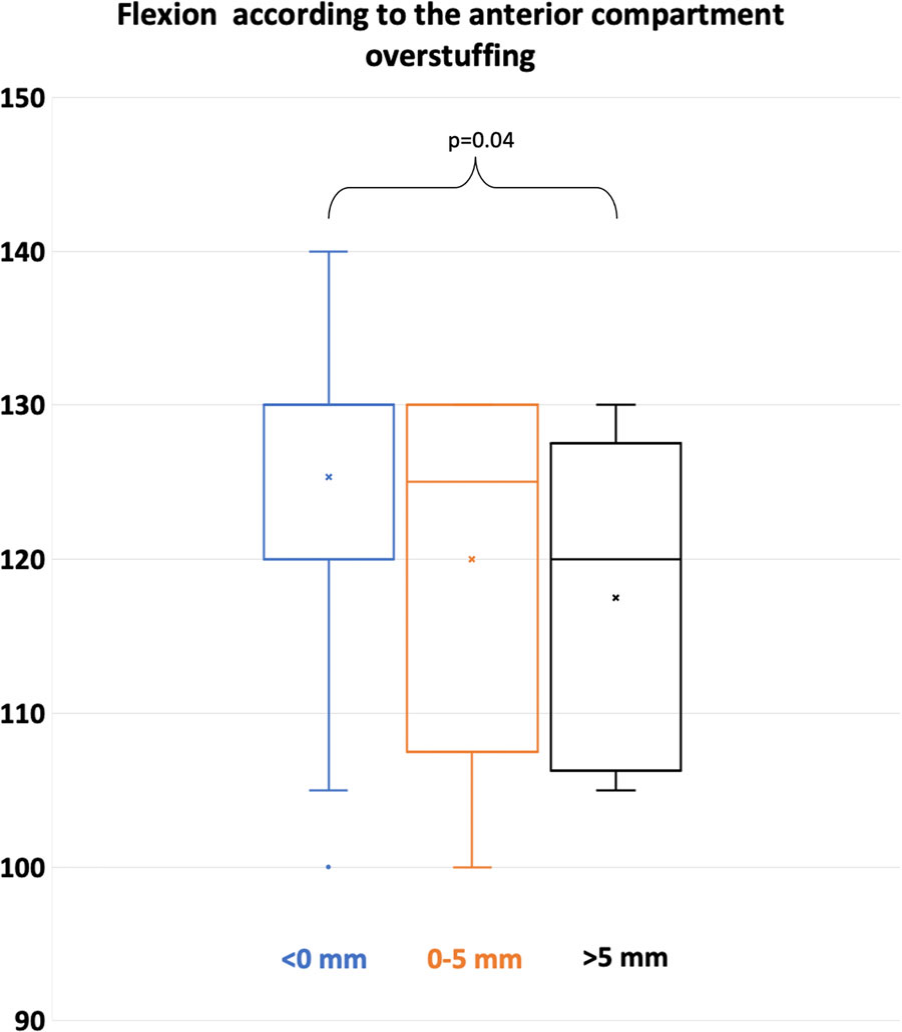
Boxplot reporting the flexion at the last follow‐up according to the anterior compartment restoration at 90° (<0, 0–5 or >5 mm).

Functional scores (knee flexion, Kujala score, FJS, KSS knee and functional scores), patient satisfaction, patellar height and patellar tilt were not significantly influenced by the anterior compartment stuffing at 0° and 30° of knee flexion. At 70°, the postoperative KSS function score decreased significantly when the overstuffing of the anterior compartment increased (*p* = 0.009). In the analysis by subgroups, at the position 90°, the flexion was significantly lower when the anterior compartment overstuffing was superior to 5 mm (117.5° ± 10°) compared to an overstuffing between 0 and 5 mm (120.0° ± 13°) and between 0 and −5 mm (125.3° ± 8°) (*p* = 0.04) (Figure 5).

## DISCUSSION

The main findings of this study were a tendency to understuff the anterior compartment after primary TKA performed with an image‐based robotic‐assisted system and functional positioning principles. There was a significant impact on the functional outcomes (KSS function score and knee flexion) when the anterior compartment was overstuffed at 70° and 90°.

Using classic mechanical instrumentation, the surgeons usually focus on the tibiofemoral joint, overlooking the patellofemoral joint's complexity by simply managing rotation and lateralisation of the femoral component. This unawareness of the anterior compartment can lead to early complications or revisions due to patellofemoral problems [15]. The combination of current prosthesis designs, which do not restore the anterior femur anatomy and the mechanical alignment contributed to the native‐prosthetic mismatch of the femoropatellar compartment [27, 29]. Indeed, the trochlea design was conceived for mechanical alignment with a trochlear axis of 6° compared to the distal condylar axis [30] and a dysplastic design [29]. Thanks to the preoperative CT scan, the image‐based robotic‐assisted system can materialise the shape of the native trochlea on the screen during the planning. The femoral implant is superimposed on the CT scan and positioned precisely in the same position as the native trochlea. Personalised alignment techniques, performed with an image‐based robotic‐assisted system, allowed better understanding and restoration of the trochlear groove than mechanical or kinematic alignment techniques [16, 22, 31]. Indeed, the mechanical alignment translated the groove furthest from the native anatomy compared to kinematic and functional alignment [31]. The kinematic alignment resulted in an unsafe coronal implant positioning in at least 13% of cases and an internally rotated femoral component beyond 3° in more than 25% of cases [31]. By contrast, the functional alignment had only 3.2% of patients outside coronal and 1.7% outside rotational safe zones [31]. Nevertheless, these personalised surgeries tend to understuff the trochlea and the anterior compartment, as reported in an in‐vitro study using an image‐based robotic system [31]. This current study described similar results: the femoral component showed a tendency for understuffing of the anterior compartment at 0°, 30°, 70° and for a moderate overstuffing at 90°.

**Figure 6:**
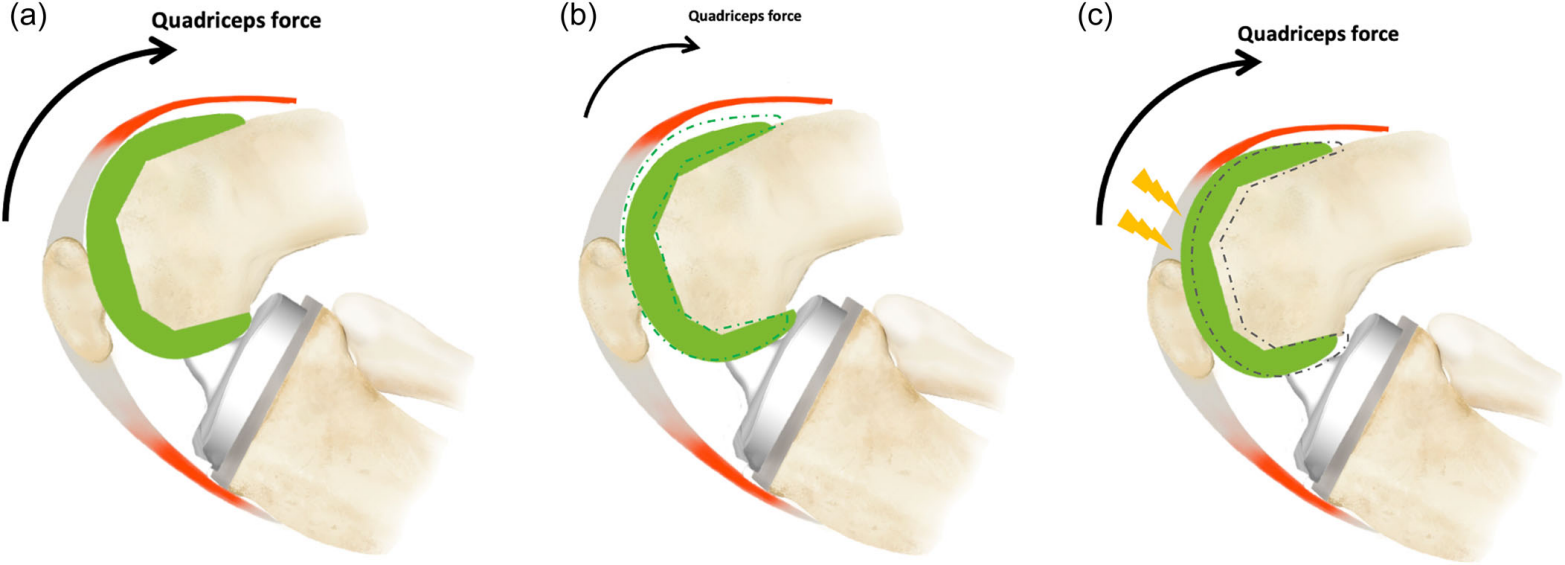
Schema of the impact on the extensor mechanism. (a) Restoration of the anterior compartment. (b) Understuffing at 30° and 70° can decrease the quadriceps force. (c) Overstuffing at 70° and 90° can increase the constraints on the extensor mechanism and decrease flexion. The dotted lines in (b) and (c) represent the normal position of the femoral implant.

The understuffing of the anterior compartment was commonly found with the functional positioning technique in mid‐flexion (at 30° and 70°). Due to the functional lengthening of the extensor mechanism [26], the patient could have a weakness in the extensor mechanism and need to increase the muscle force to generate similar movement (Figure 6). These patients could report easy fatiguability and less power when undertaking more demanding tasks [1]. In this cohort, the understuffing of the anterior compartment did not impact the functional outcomes or the patient's satisfaction. This lack of correlation was probably due to understuffing being mostly kept within the 5 mm limit, which has moderate consequences on muscle strength [23]. Another theoretic consequence might be patellar instability due to diminishing the effect of trochlea constrained in the early flexion and slack patella ligaments [25]. In this study, no patient had patellar instability after TKA. However, other authors reported that such type of atraumatic instability could be expected around 5 months and is frequently connected with femoral component flexion averaging 11° [21]. In this cohort, the approach was a subvastus approach, leading to good patellar stability without weakness of the extensor mechanism, even with moderate understuffing. Moreover, using an image‐based robotic‐assisted system allowed the restoration of the direction of the trochlea groove and the femoral rotation [31], improving the patellar tracking.

The anterior overstuffing was less common during TKA with functional positioning technique but could have more impact on the patients. This overstuffing can lead to functional shortening of the extensor mechanism and changing the surrounding soft‐tissue envelope tension [9] (Figure 6). These consequences for the patients can be anterior knee pain, extensor mechanism tensions, decreased flexion, excessive patellofemoral constraints and potentially increased component wear [9]. More than 2 mm of difference has been used as a cutoff to delineate overstuffing or understuffing for joint balancing [1]. Maniar et al. using intraoperative navigation, found a correlation between trochlear overstuffing after TKA and anterior knee pain [18]. Similar results were observed with the image‐based robotic system in this cohort. From 5 mm of anterior overstuffing, the patient could have a functional impact. The KSS function score decreased with an overstuffing of the anterior compartment at 70° and knee flexion decreased progressively with overstuffing at 90°. These observations can be due to higher constraints on the extensor mechanism, particularly the patella, with an overstuffing in deep flexion. Previous in vitro cadaveric study confirmed that with every 2 mm of patellofemoral overstuffing, 2–3° of knee flexion is lost [20]. Bracey et al. in a cadaveric study, overstuffed the patellofemoral joint progressively until the extreme measures (+6 mm, +8 mm and more) and stated that observed changes are even more significant in terms of reduced flexion, patellar shift and patellar tilt when overstuffing is ≥8 mm [4]. The overstuffing of the functionally positioned implant remained relatively uncommon, mainly occurring at 90° of flexion. A more significant number of the cases were within the 5 mm safe limit, which is the cutoff for functional impairment.

This study's findings raise the question of using standard implant designs with contemporary arthroplasty alignment techniques. Modern implant design has geometries more closely resembling the anatomic but still demonstrate ‘dysplastic’ characteristics [16]. Customised implants with personalised trochlea or specific trochlea for functional alignment could be interesting to better restore the anterior compartment and the patellar tracking.

Several limitations should be pointed out in this study. First, the mean follow‐up was relatively short. However, a 1‐year follow‐up is enough to assess postoperative anterior pain and functional outcomes after a primary TKA. This cohort will be followed for several years to evaluate the functional evolution. The results must be assessed early to detect potential bad outcomes. Second, this study was retrospective and on a small cohort. Nevertheless, the assessed clinical data were collected systematically and prospectively in the medical file. A postanalysis was performed and demonstrated a satisfying study's power. Third, evaluating the functional outcomes was subjective based on scores and autoassessment. We did not measure the quadriceps strength. Isokinetic tests are not appropriate for patients who recently operated on TKA. Fourth, we assessed only one TKA design. The shape of the trochlea varies greatly depending on the implant. Nevertheless, considering only one design allowed for homogeneity in this study. Then, the global anterior compartment restoration formula was based on the combination of all the measurements of the trochlea. This method can be simplistic but more straightforward to compare and analyse. Finally, this study concerned only varus osteoarthritic knees operated by functional positioning technique.

## CONCLUSION

Moderate anterior understuffing was frequently observed after TKA performed with functional positioning and an image‐based robotic‐assisted system. Nevertheless, this understuffing did not significantly influence the functional outcomes at the 12‐month follow‐up, particularly, the Kujala and FJSs. The overstuffing of the anterior compartment at 70° and 90° led to a reduction in KSS function score and flexion measurements at the 12‐month follow‐up and should be kept within the 5 mm limit in this cohort. Optimisation of trochlear restoration with TKA should be further explored in the future development of modern TKA.

## AUTHOR CONTRIBUTIONS

**Moussa Kafelov**: Study design; data collection; statistical analysis; literature review and manuscript writing. **Cécile Batailler**: Study design; statistical analysis; literature review; manuscript editing and supervision. **Elvire Servien**: Study design and manuscript editing. **Sébastien Lustig**: Study design; supervision and manuscript editing. All authors read and approved the final manuscript.

## CONFLICT OF INTEREST STATEMENT

S.L.: Consultant for Stryker, Smith Nephew, Heraeus, Depuy Synthes; Institutional research support from Groupe Lepine, Amplitude; Editorial Board for *Journal of Bone and Joint Surgery (Am)*. The remaining authors declare no conflict of interest.

## ETHICS STATEMENT

All procedures were performed in accordance with the ethical standards of the institutional and/or national research committee, the 1964 Helsinki Declaration and its later amendments or comparable ethical standards. Data collection and analysis were carried out in accordance with MR004 Reference Methodology from the Commission Nationale de l'Informatique et des Libertés (Ref. 2229975V0) obtained on 6 May 2023. The study was registered and filed on the Health Data Hub website.
